# Supplementary material for: Study of Reaction Parameters for the Precise Synthesis of Low-Molecular-Weight Oligosiloxanes
Source: Materials (Basel). 2025 Dec 17;18(24):5677. doi: 10.3390/ma18245677 (PMC12734515; doi:10.3390/ma18245677)
Supplement: Supplementary file 1 [file materials-18-05677-s001.zip › materials-4006904-supplementary.pdf]

materials

Supporting Information

Study of Reaction Parameters for the Precise Synthesis of Low-Molecular-Weight Oligosiloxanes

Satoru Saotome [1,2,†], Jiaorong Kuang [1,†], Yujia Liu [1,*], Takayuki Iijima [2] and Masafumi Unno [1,*]

[1] Department of Chemistry and Chemical Biology, Gunma University, 1-5-1 Tenjin-cho, Kiryu 376-8515, Japan; satoru.saotome.ma@mcgc.com (S.S.); t242a002@gunma-u.ac.jp (J.K.)
[2] Polyester Laboratory, Polymers Research & Development Center, Mitsubishi Chemical Corporation, 1, Toho-cho, Yokkaichi-shi 510-8530, Japan; takayuki.iijima.ma@mcgc.com
* Correspondence: yliu@gunma-u.ac.jp (Y.L.); unno@gunma-u.ac.jp (M.U.); Tel.: +81-27-730-1230 (M.U.)
† These authors contributed equally to this work.

Table of contents **Pages**

1. ^{1}H NMR spectra for the crude products obtained under various reaction conditions S2–8

2. MALDI-TOF MS spectra for the crude products obtained under various reaction conditions S8–12

1. ^{1}H NMR Spectra for the crude products obtained under various reaction conditions

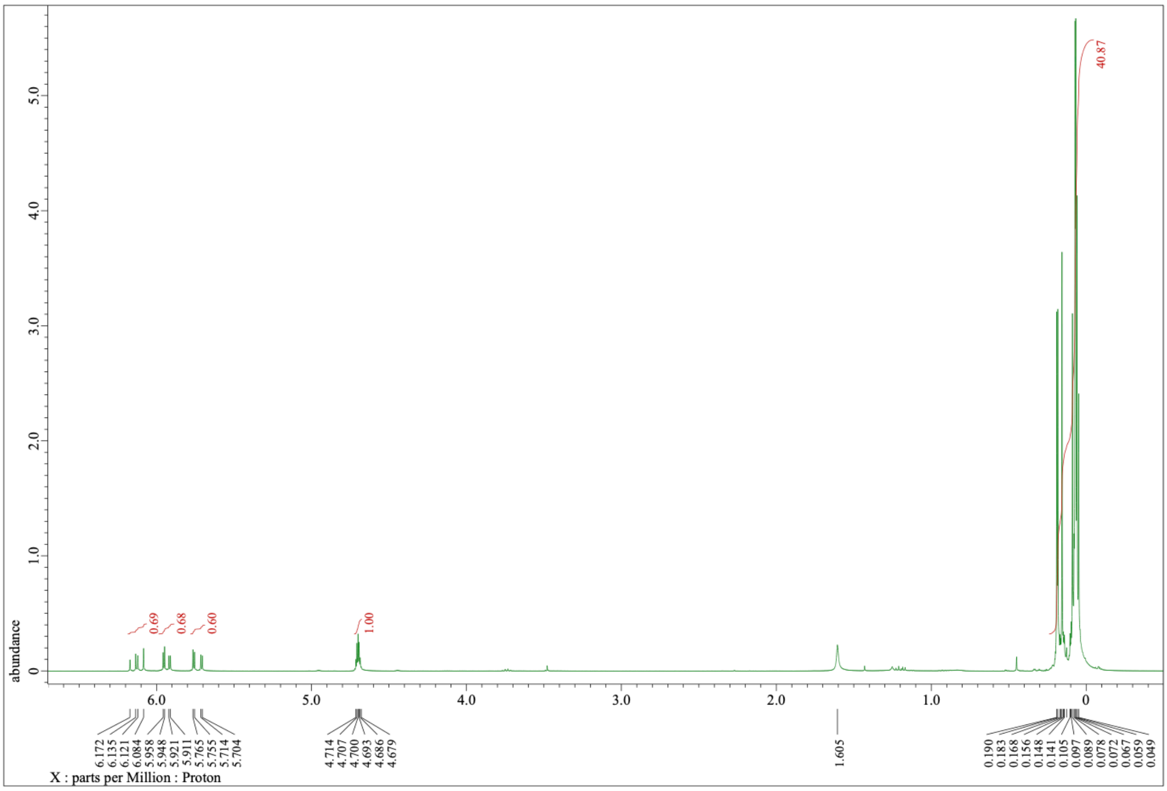

Figure S1. ^{1}H NMR spectrum for **oligosiloxane** in Table 2. Entry 1.

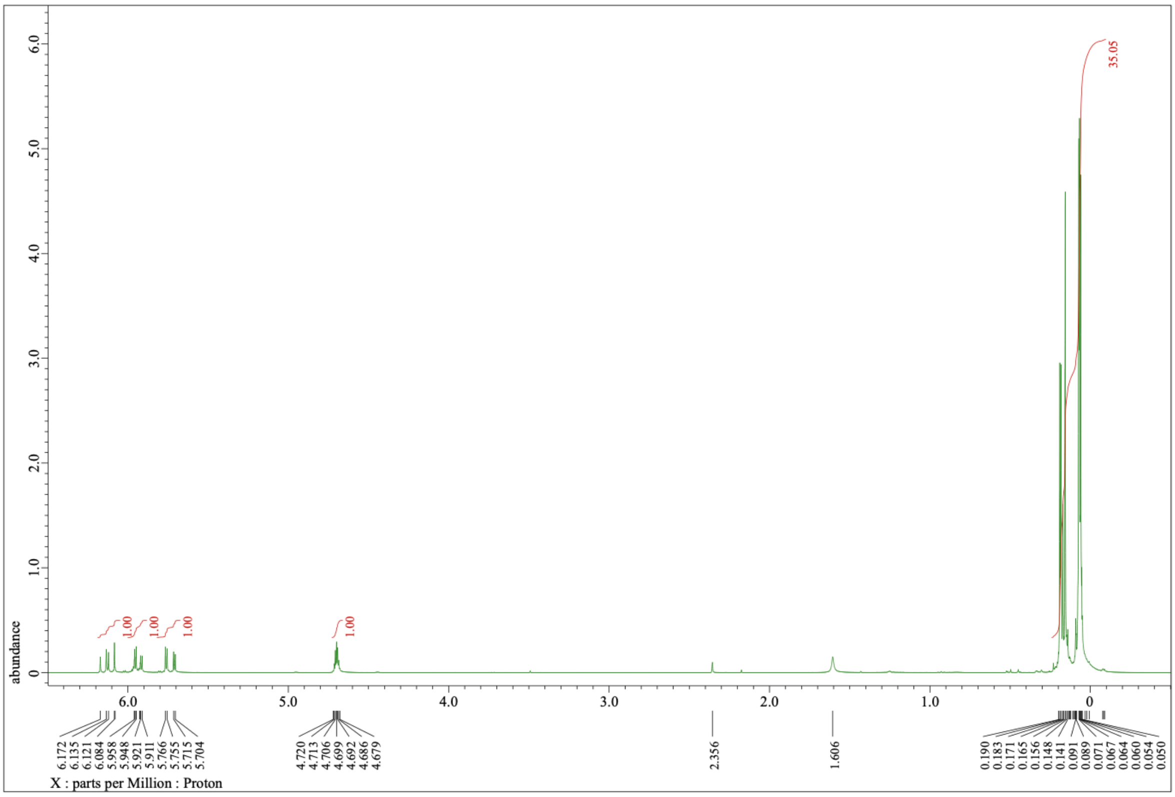

Figure S2. ^{1}H NMR spectrum for **oligosiloxane** in Table 2. Entry 2.

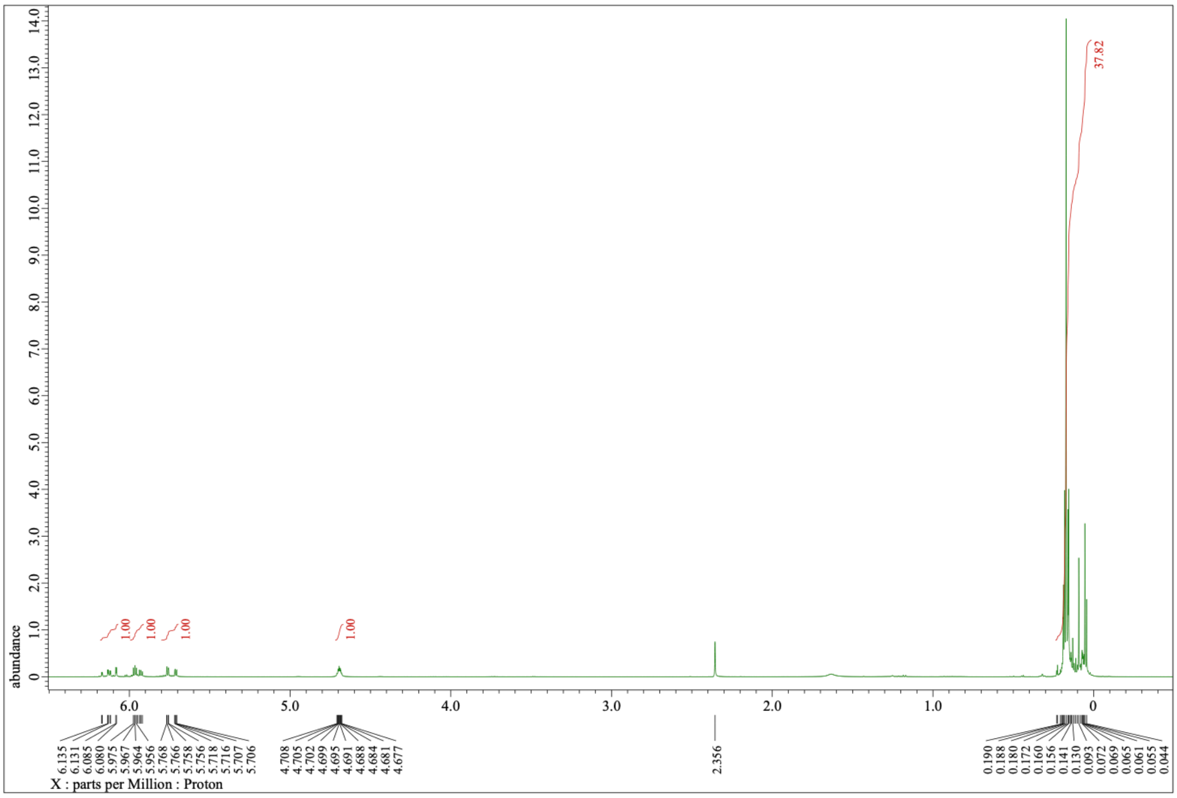

Figure S3. ¹H NMR spectrum for **oligosiloxane** in Table 2. Entry 3.

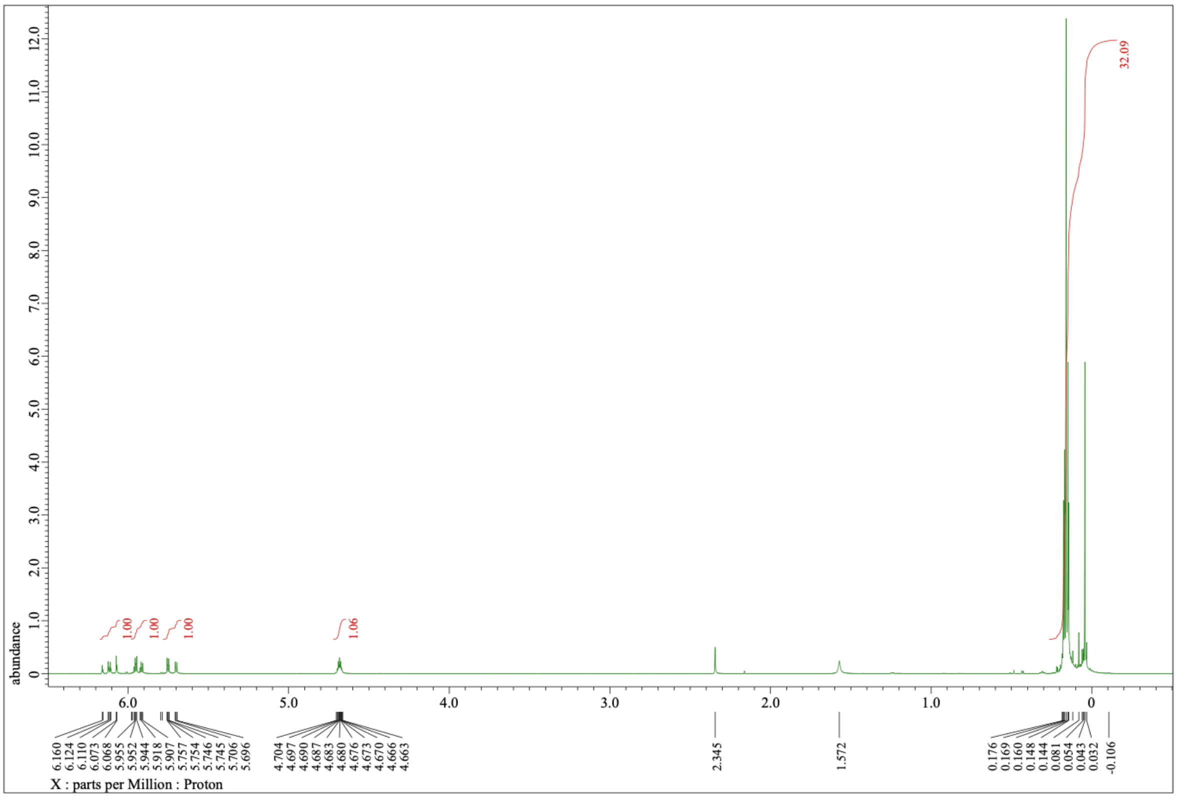

Figure S4. ¹H NMR spectrum for **oligosiloxane** in Table 2. Entry 4.

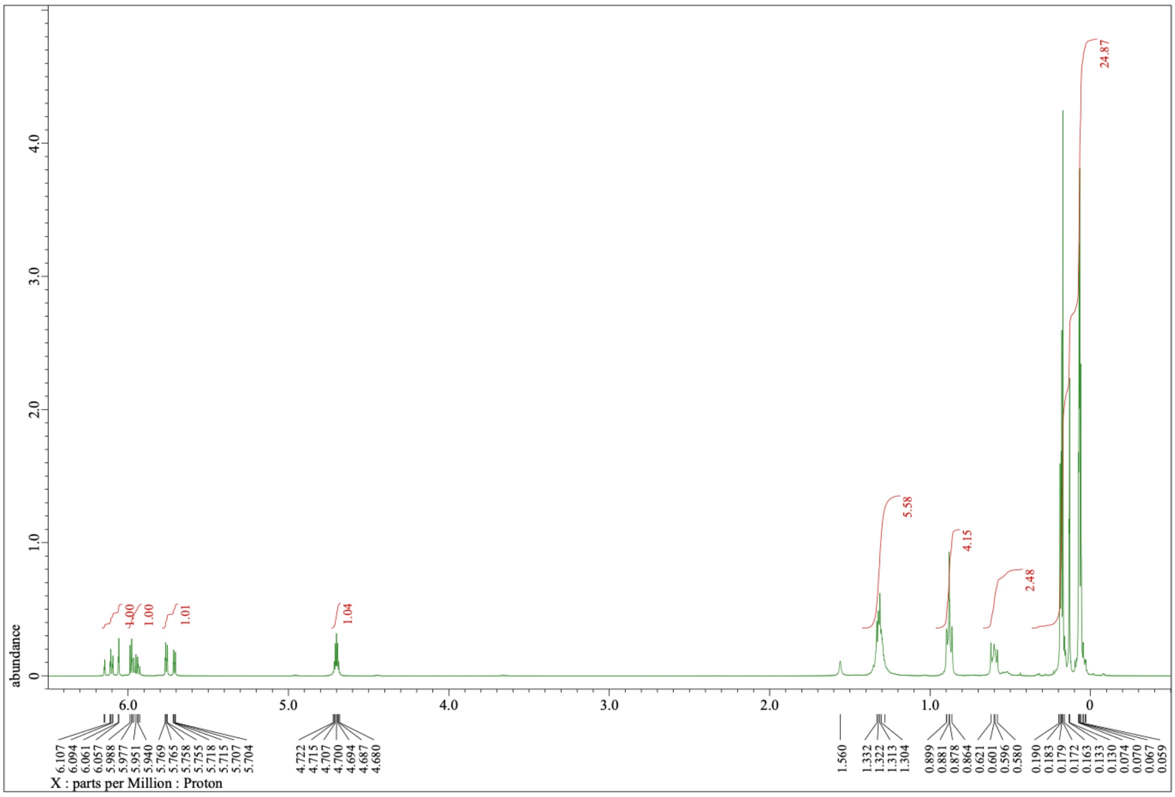

Figure S5. ¹H NMR spectrum for **oligosiloxane** in Table 3. Entry 1.

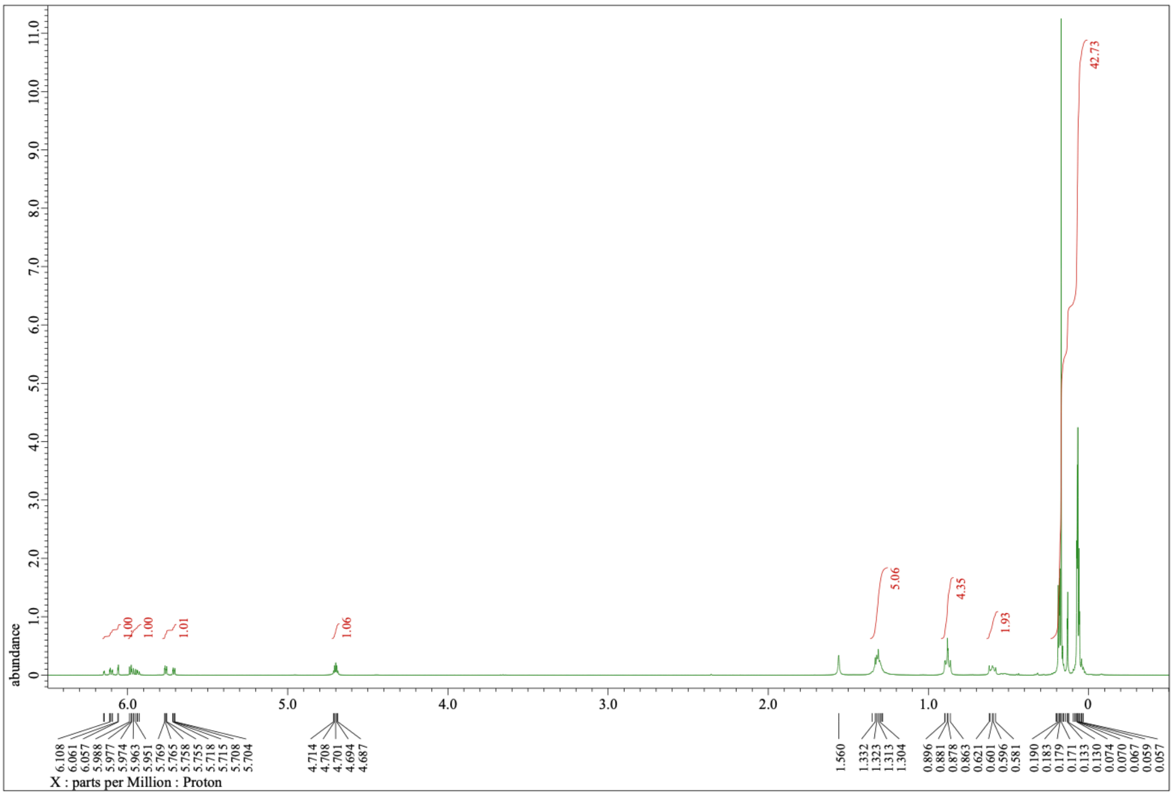

Figure S6. ¹H NMR spectrum for **oligosiloxane** in Table 3. Entry 2.

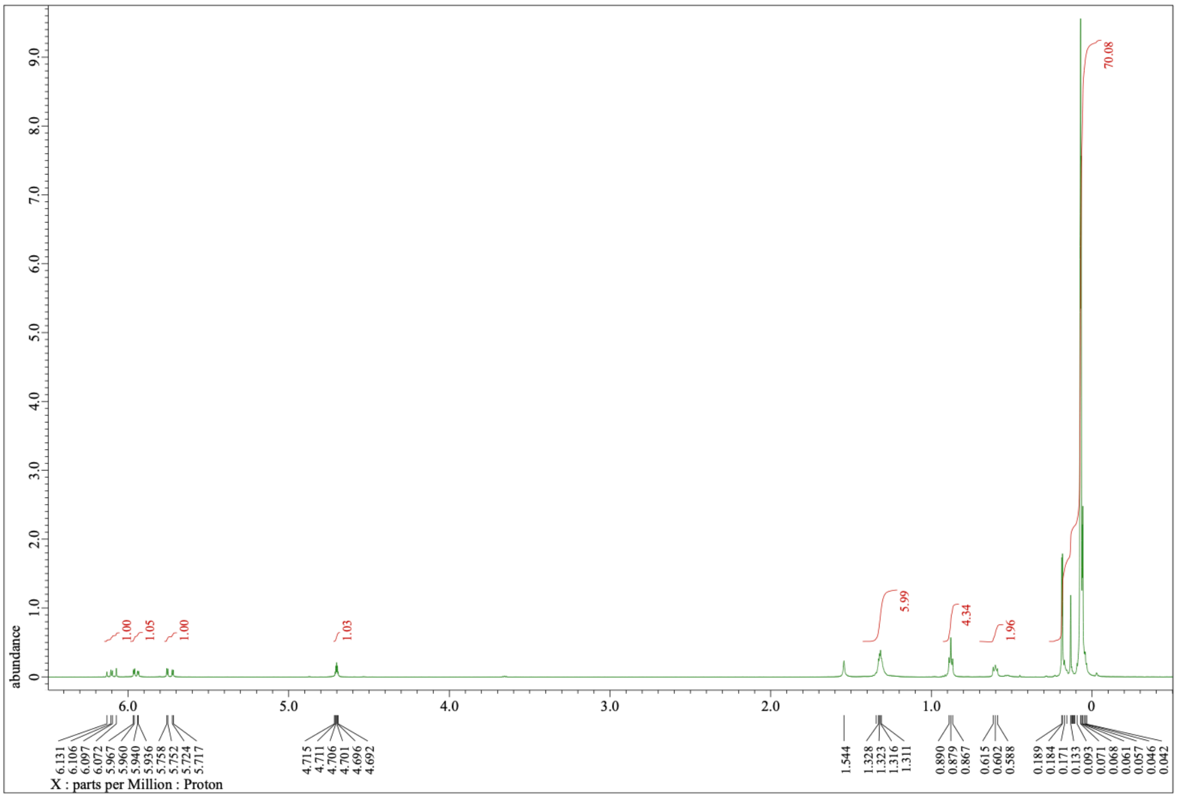

Figure S7. ^{1}H NMR spectrum for **oligosiloxane** in Table 3. Entry 3.

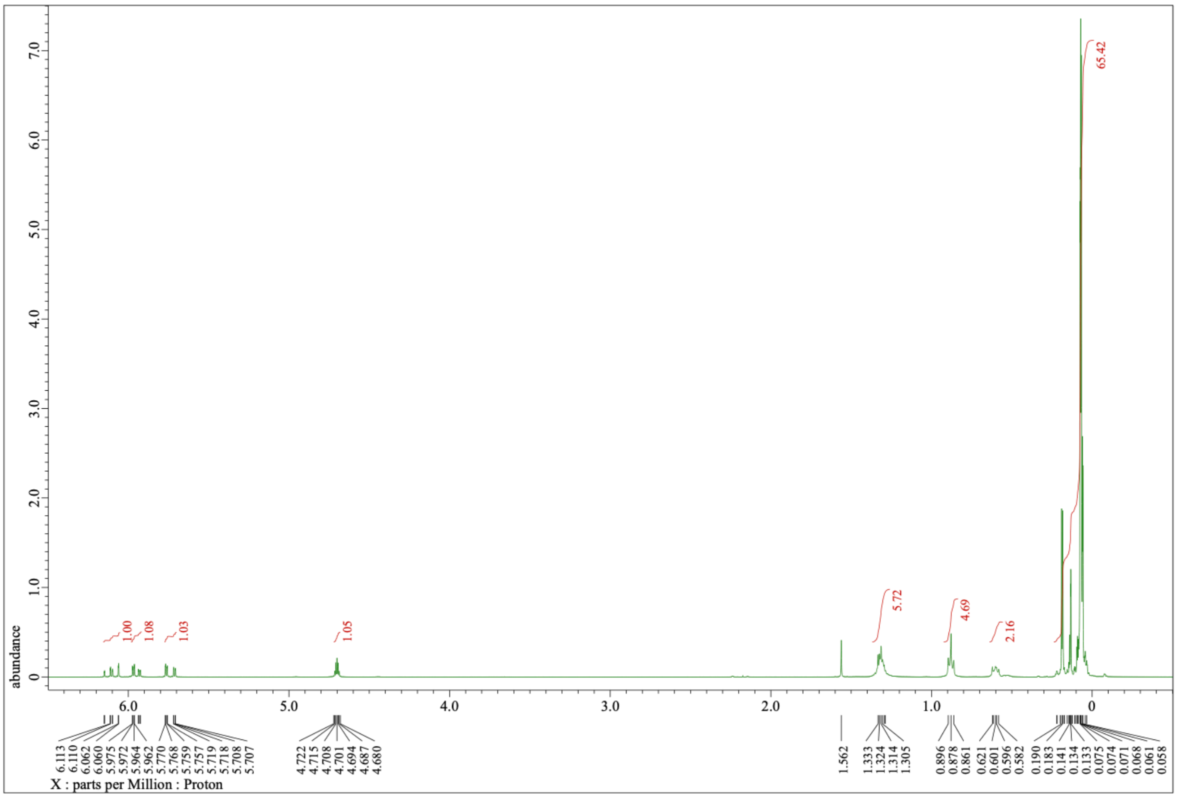

Figure S8. ^{1}H NMR spectrum for **oligosiloxane** in Table 4. Entry 1.

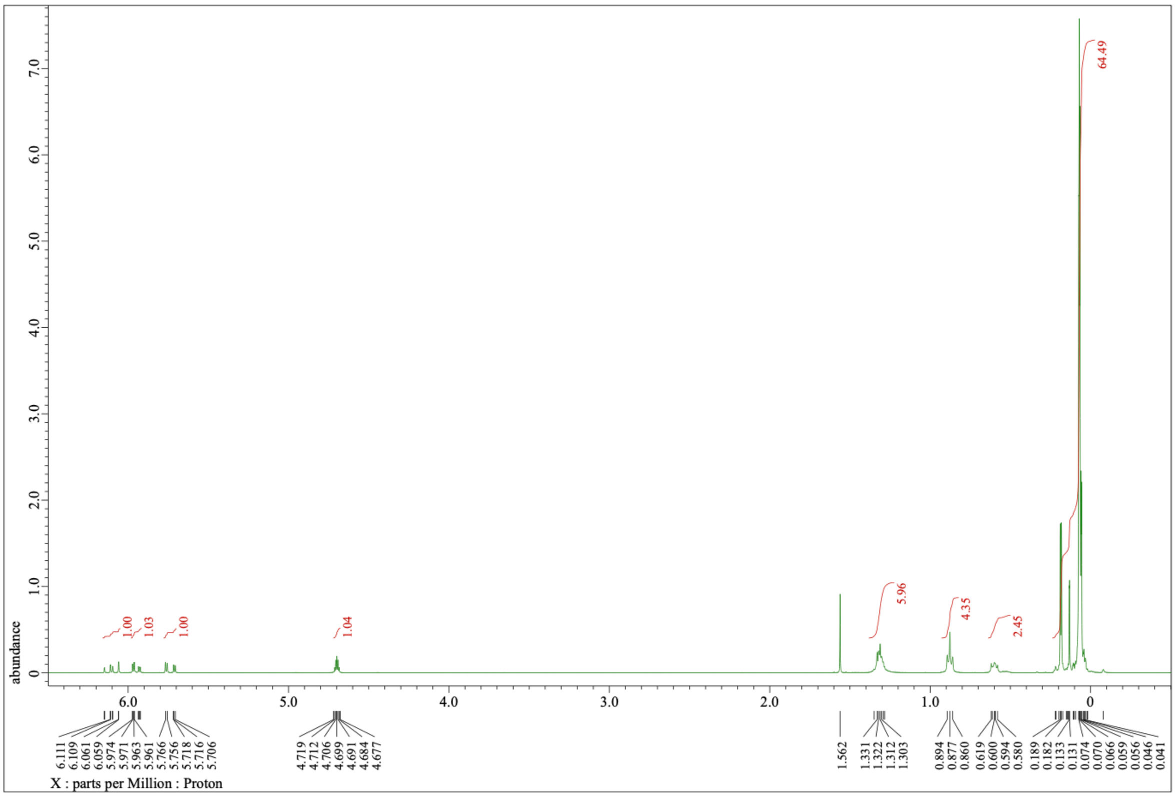

Figure S9. ^1H NMR spectrum for **oligosiloxane** in Table 4. Entry 2.

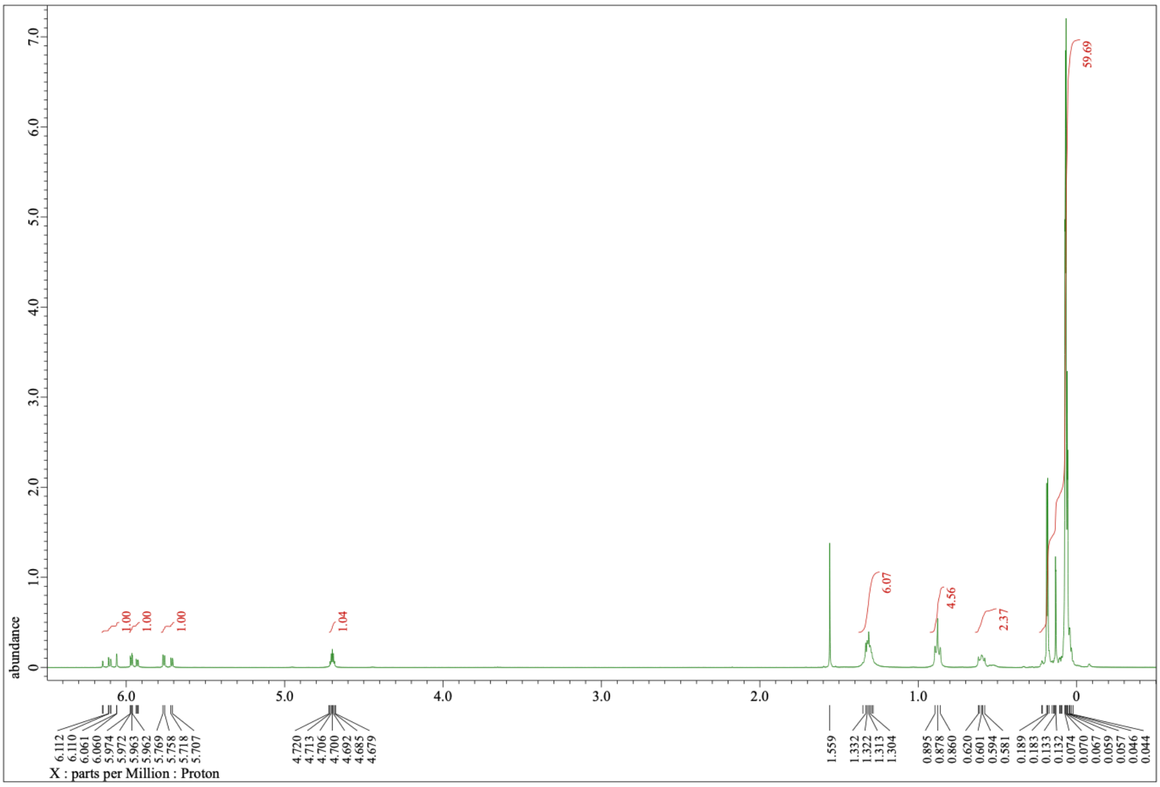

Figure S10. ^1H NMR spectrum for **oligosiloxane** in Table 4. Entry 3.

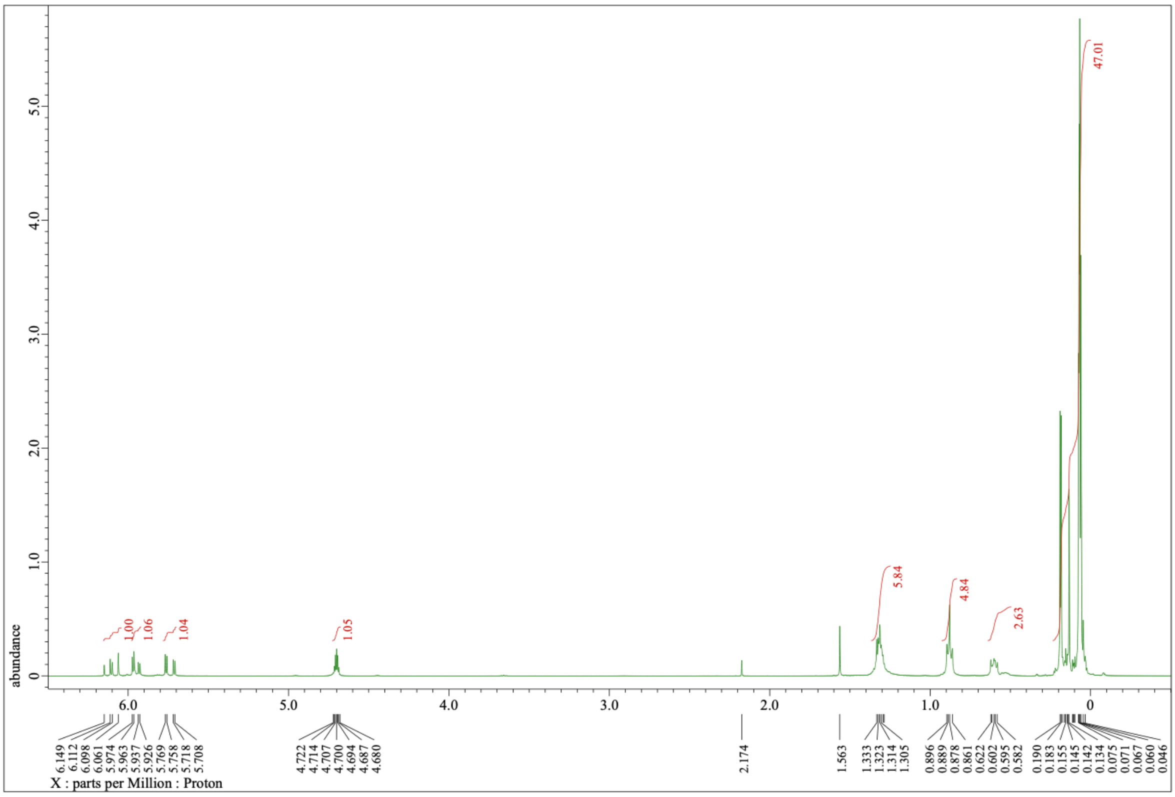

Figure S11. ^{1}H NMR spectrum for **oligosiloxane** in Table 4. Entry 4.

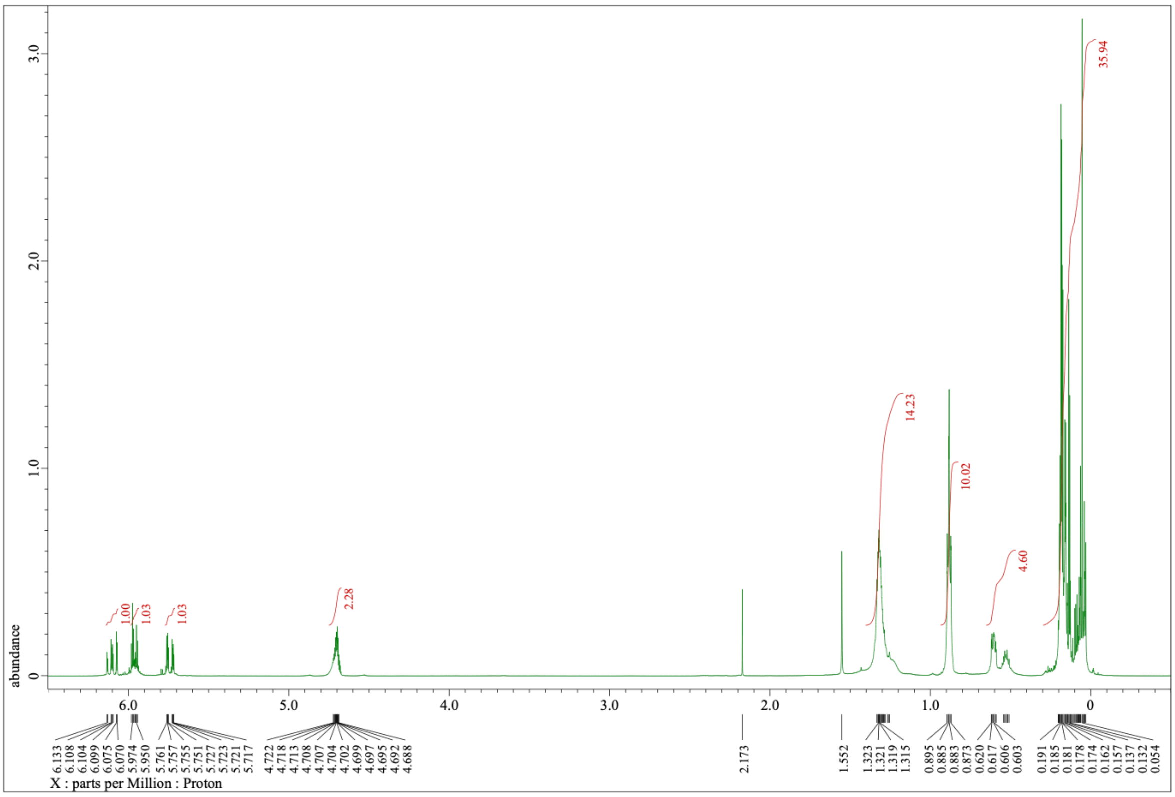

Figure S12. ^{1}H NMR spectrum for **oligosiloxane** in Table 4. Entry 5.

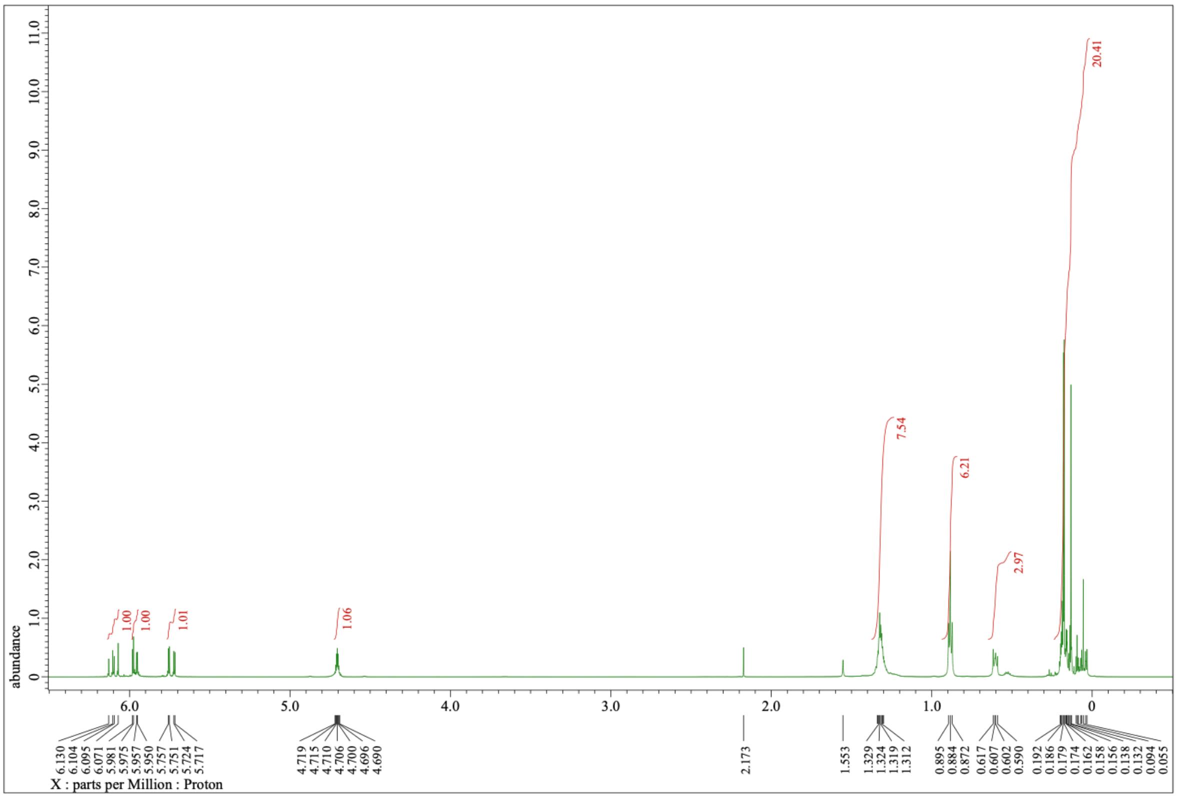

Figure S13. ^{1}H NMR spectrum for **oligosiloxane** in Table 4. Entry 6.

2. MALDI-TOF MS Spectra for the crude products obtained under various reaction conditions

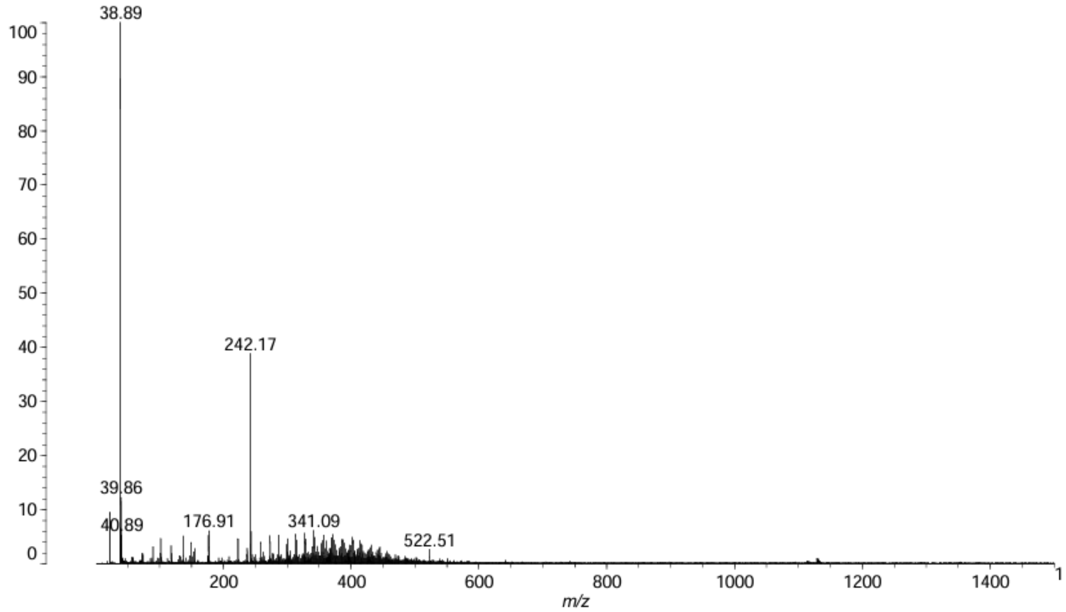

Figure S14. MALDI-TOF MS spectrum for **oligosiloxane** in Table 3. Entry 1.

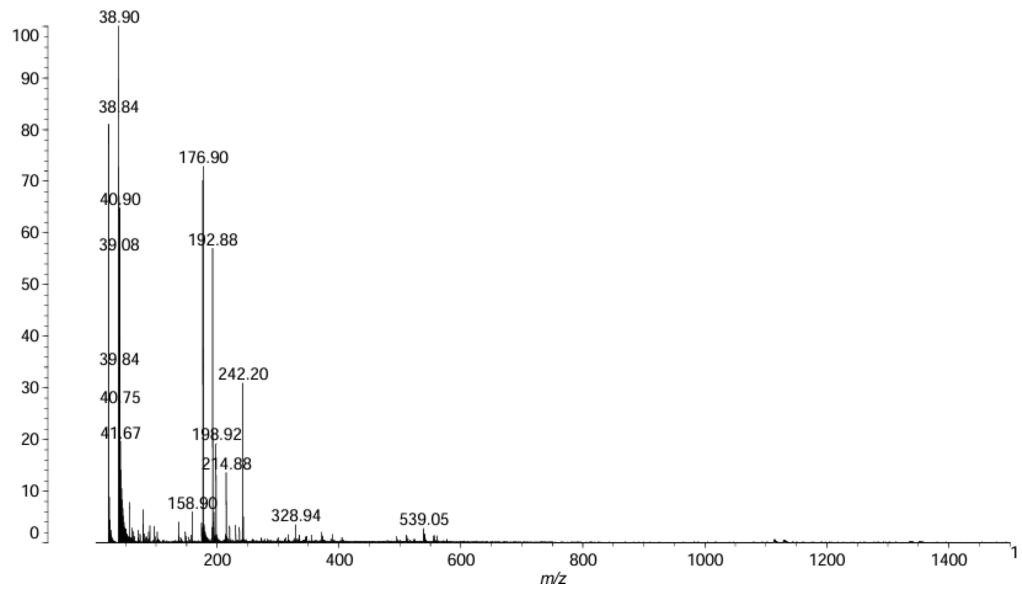

Figure S15. MALDI-TOF MS spectrum for **oligosiloxane** in Table 3. Entry 2.

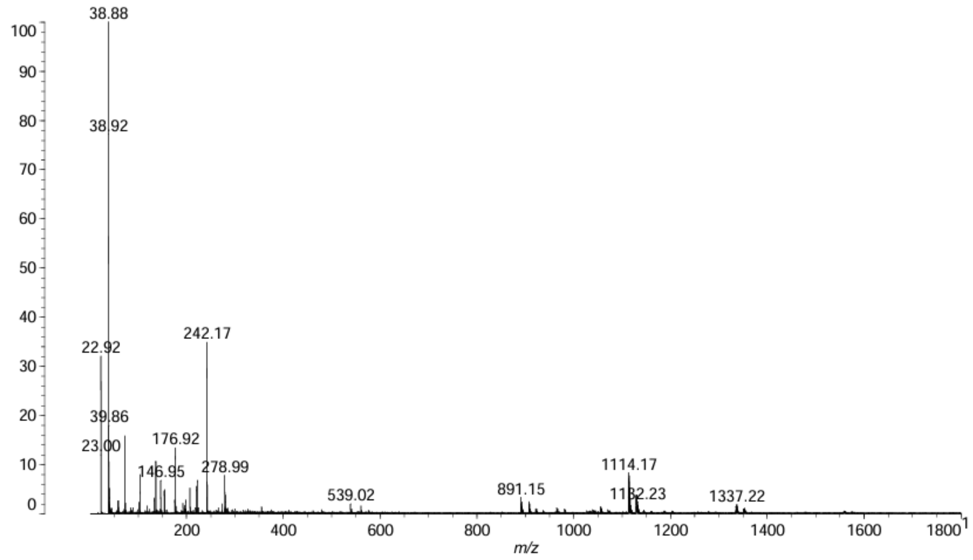

Figure S16. MALDI-TOF MS spectrum for **oligosiloxane** in Table 4. Entry 1.

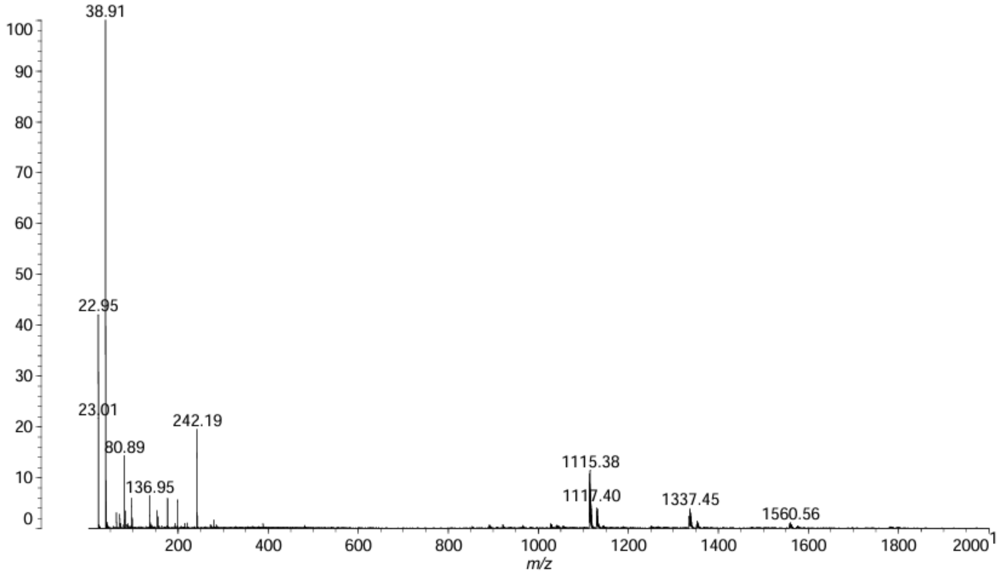

Figure S17. MALDI-TOF MS spectrum for **oligosiloxane** in Table 4. Entry 2.

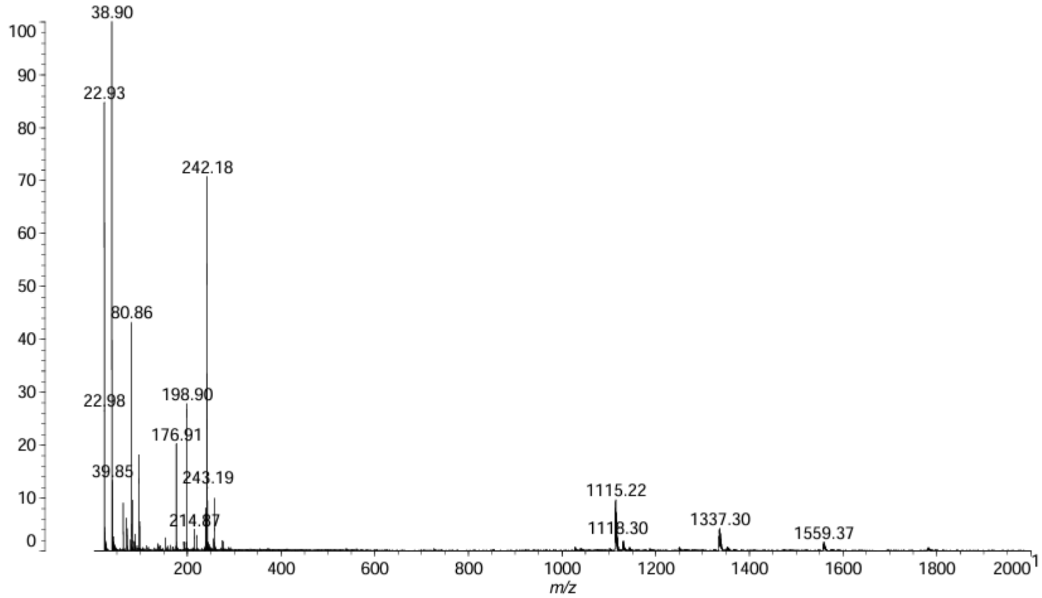

Figure S18. MALDI-TOF MS spectrum for **oligosiloxane** in Table 4. Entry 3.

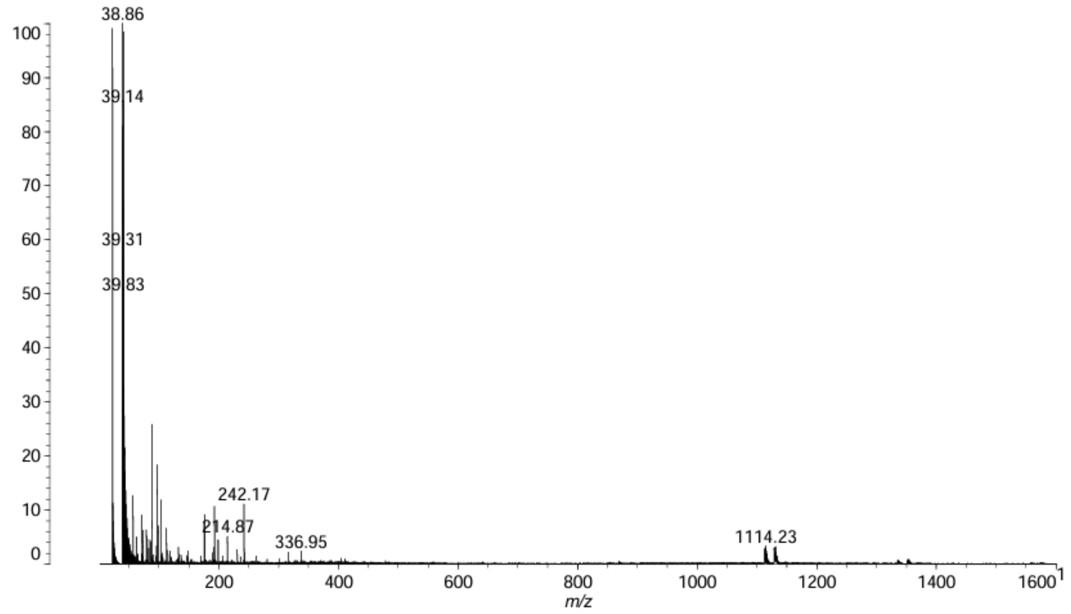

Figure S19. MALDI-TOF MS spectrum for **oligosiloxane** in Table 4. Entry 4.

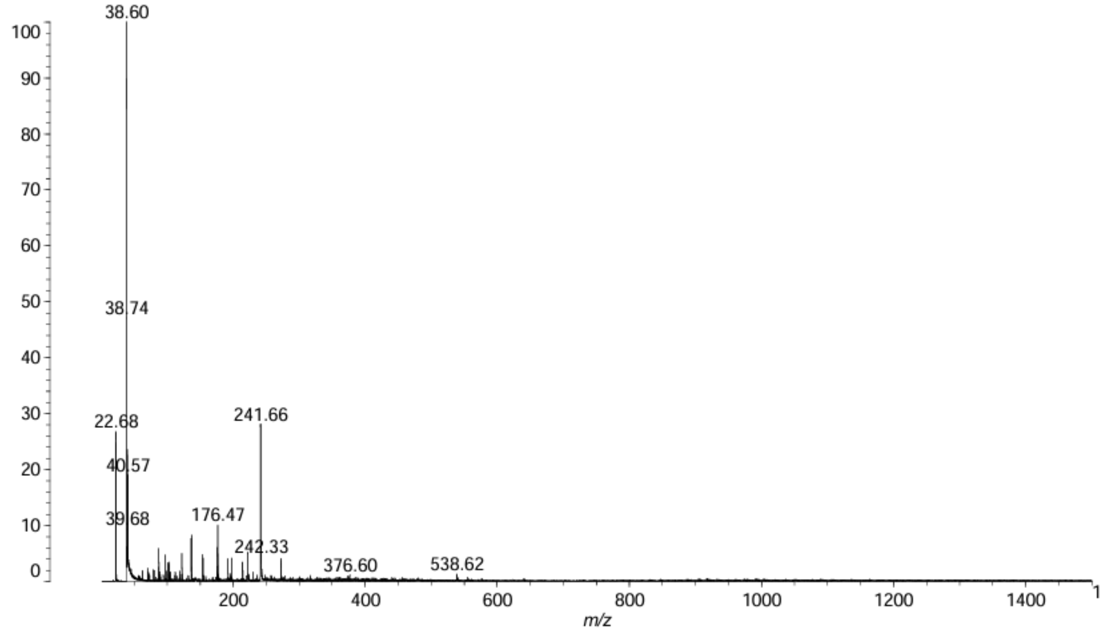

Figure S20. MALDI-TOF MS spectrum for **oligosiloxane** in Table 4. Entry 5.

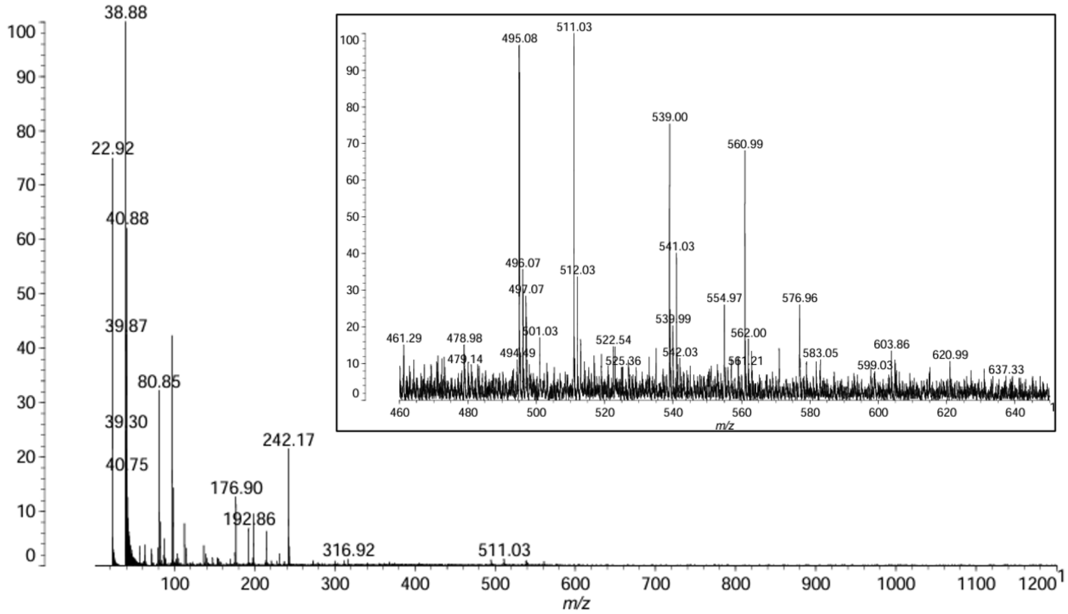

Figure S21. MALDI-TOF MS spectrum for **oligosiloxane** in Table 4. Entry 6.
